# Could driving help us to “see better”? A comparative assessment of saccadic efficiency, visual speed, and attention

**DOI:** 10.1186/s12886-024-03349-1

**Published:** 2024-02-27

**Authors:** Andrés Gené-Sampedro, Francisco Alonso, Javier Gene-Morales, Pedro Lourenço Monteiro, Sergio A. Useche

**Affiliations:** 1 Research Institute on Traffic and Road Safety (INTRAS), University of Valencia, Valencia, Spain; 2 Department of Optics and Optometry and Vision Sciences, University of Valencia, Valencia, Spain; 3 Faculty of Psychology, University of Valencia, Av. Blasco Ibáñez 21, Valencia, 46010 Spain; 4 Prevention and Health in Exercise and Sport (PHES) research group, Department of Physical Education of Sports, University of Valencia, Valencia, Spain; 5 Department of Physics, University of Beira Interior, Covilhã, Portugal; 6 CICS (Health Sciences Research Centre), University of Beira Interior, Covilhã, Portugal

**Keywords:** Oculomotricity, Reading, Processing, Distractors, ADEMd, UFOV, VF-14

## Abstract

**Background:**

This study aimed at comparing drivers’ and non-drivers’ results in the Adult Developmental Eye Movement with Distractors test (ADEMd) and the Useful Field of View test (UFOV).

**Methods:**

One hundred and twenty Spaniards (mean age 50.90 ± 17.32 years) without eye disease voluntarily participated in this cross-sectional descriptive study. Participants in a single experimental session completed a questionnaire on sociodemographic, health, eyesight, and driving information. They also performed the ADEMd and UFOV tests randomly following standardized protocols. The ADEMd is a visual-verbal test that measures saccadic efficiency and visual attention. Brown-Forsythe (B–F) tests with Games-Howell post-hoc adjustments were conducted to assess differences between groups. Groups were formed according to sex, age (young adults, adults, and older adults), and driver/non-driver for further analysis. Additionally, associations between dependent variables were assessed through Spearman’s correlations.

**Results:**

Drivers obtained significantly better results in the ADEMd compared with non-drivers. Non-significant differences between drivers and non-drivers were encountered in the UFOV. Additionally, significant differences were observed between sexes and age groups. It is worth highlighting that non-driver’s age significantly correlated with worse ADEMd performance (*rho* = .637 to .716). This correlation was non-significant in drivers. Similarly, reading hours significantly correlated with better ADEMd performance in non-drivers (*rho* = − .291 to − .363), but not in drivers. The only significant correlations between ADEMd and UFOV tests were found in drivers (*rho* = .307 to .410).

**Conclusion:**

Considering all the discussed results, it could be hypothesized that the driving task promotes abilities, such as oculomotor and cognitive function, which are relevant for the performance in the ADEMd. However, this hypothesis is based on correlational outcomes and further studies should causally assess this possible relation.

## Background

Road safety is still a major concern as one of the leading causes of death worldwide [1]. In this sense, the human factor (including mental and physical health) has been recognized as a key factor in conditioning road crashes and driving performance [2, 3]. To control this, apart from educating drivers [4, 5] and monitoring behavioral traits [6–8], visual competencies and, more specifically, ocular movements play a relevant role in road safety [9–13].

Apart from the ocular competencies assessed in driving examination centers (e.g., visual acuity and refraction errors), ocular movements (saccades, fixations, and pursuits), visual attention, and processing are crucial for proper driving and could, therefore, provide complete information on the apt ability of a person to drive [14–16]. Numerous devices are used to measure ocular movements in driving experiments [2, 6, 8, 15]. However, the devices to measure this are expensive and difficult to implement. Additionally, tests such as the Useful Field of View (UFOV) measure visual attention, processing, and susceptibility to distractions [17, 18]. This test, although associated with driving performance and involvement in road crashes [18–20], does not allow the participant to perform ocular movements (see “UFOV” section).

Nevertheless, to date, efficient, inexpensive, simple-to-implement, and validated visual-verbal tests exist, such as the Developmental Eye Movement (DEM) test [21] and its version for adults, the Adult Developmental Eye Movement test (ADEM) [22], both of them designed for simple clinical testing of saccadic performance and automaticity through rapid number-naming [23]. Among other purposes, the ADEM has recently been used to compare oculomotor efficiency and visual attention in drivers and non-drivers [24], as suggested by previous expert literature [23]. Recently, a modification of the ADEM consisting of a new sheet that includes distractors, the Adult Developmental Eye Movement with Distractors test (ADEMd, see “ADEMd” section) has been validated [25] to overcome the limitations of the original version and to increase the demands in visual processing and attention. Therefore, the question arises as to whether this new version can be useful to differentiate between drivers and non-drivers.

The ADEMd and the UFOV are visual tests that share certain features in common but differ in others. In particular, both tests require high processing ability and attention to detect and identify targets (see “Methods” section). The main difference between both tests is that, on the one hand, the UFOV requires minimum head or eye movements, and the distractors are part of the background. On the other hand, the ADEMd requires eye movements typically present in reading with minimum head movement and includes distractors within the targets. During driving, visual information can be recognized, analyzed, and processed through optimal eye movements (saccades, fixations, and pursuits) and attention, allowing the driver to understand, organize, and act within a dynamic changing environment [10, 13, 26, 27].

Bearing in mind that drivers constantly use saccades and attention for proper performance [16], a certain oculomotor and cognitive training effect could be expected to make drivers obtain better results in the ADEMd compared to non-drivers.

Therefore, the purpose of the present study was to compare drivers’ and non-drivers’ results in the Adult Developmental Eye Movement with Distractors test (ADEMd) and compare the results with the Useful Field of View (UFOV) test. We hypothesized, regarding this study aim, that drivers would present better performance (less time) in the ADEMd test and UFOV.

## Methods

This cross-sectional study aimed at evaluating the usefulness of a visual-verbal test that measures saccadic efficiency and visual attention (ADEMd) in differentiating between drivers and non-drivers and, therefore, analyzes its potential to be included within the driver examination procedures. For this purpose, the results were compared with the outcomes obtained in the UFOV, which has been recognized as potentially useful in the prediction of visual problems associated with driving risk (as mentioned in the introductory section).

As for ethical issues, it is worth pointing out that all subjects were voluntarily enrolled in the study. Each subject signed an informed consent statement before starting the study and was free to withdraw at any time. The study was conducted in conformity with the Code of Ethics of the World Medical Association (Declaration of Helsinki), and ethical authorization was provided by the Research Ethics Committee for Social Science in Health of the INTRAS (University Research Institute on Traffic and Road Safety) of the University of Valencia (IRB approval number HE000171016).

All data retrieved were confidential and anonymous; the questionnaires and tests were designed and applied to ensure this. Each participant was anonymized using an alpha-numeric code. No potential risks to the integrity of the subjects were identified.

### Procedure

Following the research protocol, all key study-related procedures (i.e., data collection setting) took place under the same environmental conditions and by the same trained optometrists and ophthalmologists. A 1:1 ratio of researcher-subject was always maintained (at least). The full experimental session had a mean duration of 40 min. All tests were performed binocularly and using optical correction if the participant normally used them (e.g., glasses or contact lenses). Potential biasing factors such as testing position and distance, room lighting (always constant at approximately 250 lux), and distracting factors (e.g., external noise or mobile phones) were controlled through rigorous surveillance of the research staff members to avoid measurement gaps.

Before starting, all the subjects were informed about the aims and procedures of the investigation. At this point, they underwent a full optometric examination and completed a questionnaire on sociodemographic, health, eyesight, and driving information to certify their validity, gather descriptive data, and characterize the independent variables. All participants also performed the clock-drawing cognitive test [28] and a validated version of the Mini-Mental State Examination (MMSE) for the Spanish population [29, 30] to exclude potential visual and/or cognitive impairment.

After confirming the eligibility of each participant and a thorough explanation, the ADEMd and UFOV tests were performed in random order to measure the dependent variables (see “ADEMd” and “UFOV” sections). All the instruments (questionnaires and tests) employed in the study are described in detail within the following subsections.

#### Main questionnaire

Previous research [16, 24, 31, 32] was consulted to design the initial questionnaire. The questionnaire was presented in Spanish and filled out by hand. The questionnaire was composed of thirty-three questions divided into four sections (sociodemographic, health, eyesight, and driving information), and was completed in around 10 min. It is important to mention [9, 16, 33] that all the data gathered in this section were self-reported. Self-reported health information has been related to actual disability and psychosocial and chronic health issues, including ophthalmic conditions [34, 35].

- *Sociodemographic information*: Age, sex, and academic achievements were included in this section to characterize the sample and assess correlations with the study-dependent variables. The educational level was ordinarily coded into 1 = Secondary education or lower, 2 = Middle education completed (baccalaureate or equivalent), and 3 = University studies or equivalent.
- *Health information*: The questions related to health, although answered by all the subjects, were based on ophthalmological research for older patients [36]. Participants were asked about suffered conditions (diabetes, hypertension, thyroids, anemia, and others), involvement in drug treatment, changes to diet, sleep, medication, trauma, or stress within the last three months. The quality of life of the participants was enquired about through a Likert ordinal scale from 0 (very poor) to 4 (excellent).
- *Eyesight-related information*: In this section, subjects were first asked about any suffered ocular conditions, such as strabismus, elevated intraocular pressure, and current or past ocular surgery. Then, participants self-reported how many hours per week they spend reading (1 = less than three hours; 2 = from three to six; 3 = from seven to fourteen; 4 = from fifteen to twenty-one; 5 = more than twenty-one hours per week) and the quality they perceived in reading and their general sight (0 = very poor; 1 = poor; 2 = average; 3 = good; 4 = excellent). The Visual Function Index Test (VF-14) [37] was conducted to end this section. Fourteen questions related to fourteen daily activities such as driving, reading, watching television, and cooking are included in this test, with potential answers being 0 (unable to do it), 1 (much difficulty), 2 (some difficulty), 3 (not much difficulty), and 4 (no difficulty). The final score is calculated by averaging all the answers and multiplying the value by 25 to obtain a value out of 100. The VF-14 test has previously been validated in drivers [38] and is strongly correlated with visual satisfaction but not with visual acuity or health [39–42]. The Spanish version of the test was used, as it is reliable, valid, and sensitive to change [43].
- *Driving information*: This section enquired about the participants’ driving experience (years being a driver, with 0 = non-driver; 1 = less than 5 years; 2 = between 5 and 15 years; and 3 = more than 15 years). Kilometers driven per week during the previous year were enquired about. This value was extrapolated to kilometers driven per year and scaled as 0 = non-driver, 1 = under 2500 km/year, 2 = between 2500 and 10,000 km/year, and 3 = more than 10,000 km/year. Finally, participants rated between 0 (none) and 4 (much) the difficulty they perceive in driving during the day and at night.

#### ADEMd

The Adult Developmental Eye Movement test with distractors (ADEMd) consists of four sheets of size 11 Times New Roman font numbers presented in DIN-A4, equivalent to a Snellen resolution of 20/80 when presented at 40 cm. Two of the sheets contain 40 numbers (two digits) vertically aligned and distributed across two columns widely separated (20 number spaces separation) and twenty rows (two numbers per row), and the other two sheets contain the same 80 numbers horizontally aligned and distributed across ten columns and sixteen rows (five numbers per row unevenly distributed). The numbers of the horizontal sheets are presented in a different order compared to the vertical sheets; both horizontal sheets include the same numbers distributed in the same order.

**Fig. 1:**
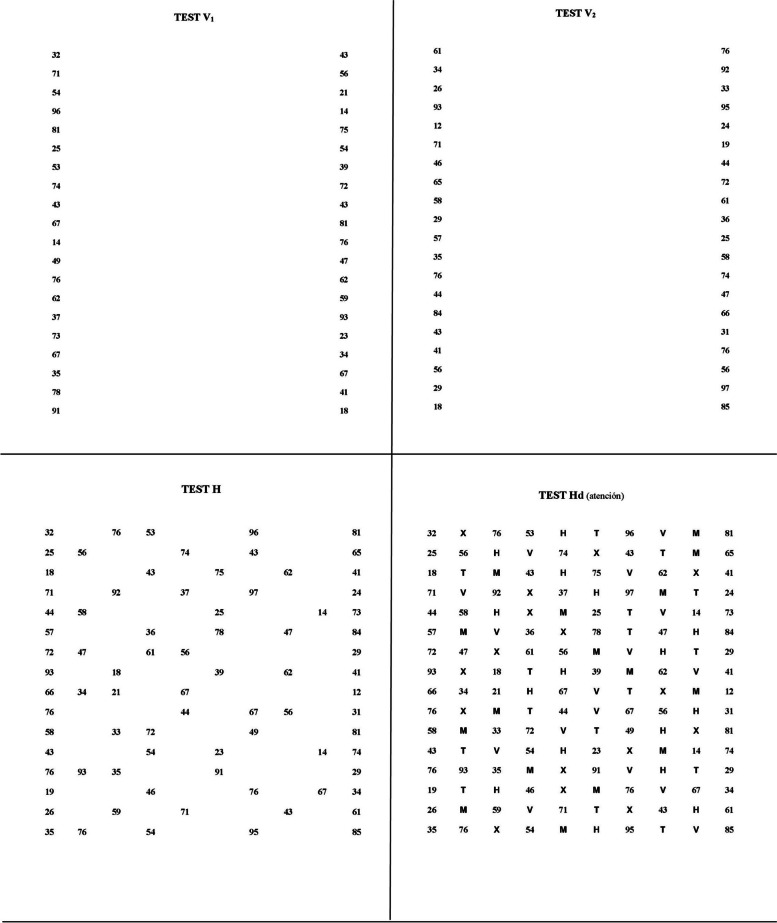
Adult Developmental Eye Movement test with distractors (ADEMd) test vertical sheet 1 (upper left), vertical sheet 2 (upper right), horizontal sheet (bottom left), and horizontal sheet with distractors (bottom right)

 The extremes of the numbers of the columns of the vertical sheets subtend an angle of 20.9º vertically and 17.3º horizontally when viewed at 45 centimeters. The vertical angular separation between numbers is 1.2º, which is an area that falls within the central retina. The extremes of the numbers of the lines of the horizontal sheets subtend an angle of 17.1º vertically and 17.4º horizontally when viewed at 45 centimeters. The horizontal angular separation between characters varies from 1.91º to 9.78º, with a mean value of 4.39 ± 1.93º. One of both horizontal sheets includes the letters “H”, “M”, “T”, “V”, and “X” between the numbers filling all the free spaces (five letters per row). All four sheets can be seen in Fig. 1.

Each subject (participant) was sitting upright on a chair, and the test was placed on a stand on a table. The subject was positioned 40–50 cm away from the test (mean of 42.7 ± 3.1 cm). Each participant had to read the numbers out loud as fast as possible, from top to bottom (vertical sheets) and from left to right (horizontal sheets), with the instruction not to stop when an error occurred. As explained below, a clinician recorded the speech with a tape recorder to evaluate the test performance ex-post. The time needed to read both vertical sheets (40 numbers each) was added up to obtain a single vertical value (time needed to read 80 numbers), as explained below. In the horizontal sheet with distractors, only the numbers had to be named, avoiding reading the letters. Therefore, errors consisted of omission, addition of numbers, or naming letters. The scoring of the test was calculated considering the following guidelines [25]:

1. Adjusted vertical time (V_adj_), which is a measure of the naming speed and automaticity, was calculated in seconds as: *V*_*adj*_ = V*80/(80-omissions + additions), where V (vertical time) are the seconds needed to read both vertical sheets.
2. Adjusted horizontal time (H_adj_), which is an indirect evaluation of the pursuits and saccades involved in reading combined with number-naming automaticity, was also calculated in seconds as: *H*_*adj*_ *= H*_*1*_ *80/(80-omissions + additions), where H_1_ (first horizontal time) are the seconds needed to read the first horizontal sheet (without distractors).
3. Adjusted horizontal time with distractors (Hd_adj_), which indirectly measures the same constructs as H_adj_ but adds the requirements of divided attention to name numbers and not letters, was calculated in seconds as: *Hd*_*adj*_ *= H*_*2*_ *80/(80-omissions + additions + letters), where H_2_ (second horizontal time) are the seconds needed to read the second horizontal sheet (with distractors).
4. Ratio H_adj_ / V_adj_, this ratio compares horizontal (oculomotor control with automaticity) and vertical (naming speed and automaticity) levels.
5. Ratio Hd_adj_ / V_adj_, which compares the horizontal attentional task incorporating the distractors (oculomotor control with automaticity and divided attention) with the vertical levels (naming speed and automaticity).
6. Ratio Hd_adj_ / H_adj_, which compares the attentional horizontal incorporating the distractor task (oculomotor control with automaticity and divided attention) with the horizontal (oculomotor control with automaticity) levels.

As presented in the validation of the ADEMd [25], reliability assessments showed high consistency between the time needed to read the first and second vertical sheets (Spearman-Brown coefficient = .98), between the time needed to read the first and last 40 numbers of the horizontal sheet (Spearman-Brown coefficient = .95), and between the time needed to read the first and the last 40 numbers of the horizontal with distractors sheet (Spearman-Brown coefficient = .96). The internal consistency between the sheets was also excellent (V_adj_ - H_adj_: .96; V_adj_ - Hd_adj_: .93; H_adj_ - Hd_adj_: .95). Finally, test-retest reliability (intraclass correlation coefficient) ranged between .81 and .97 in all the four sheets.

#### UFOV

The Useful Field of View test (UFOV, Visual Awareness Inc., Birmingham, AL, USA) is a visual-manual test performed with computer software with the subject seated at a distance of between 45 and 61 cm. The completion of the test took around fifteen minutes. This test, performed binocularly and based on rapid visual processing, assesses the useful information that can be obtained with no eye or head movements with sight fixed in a central picture. The test quantifies (in milliseconds) the quality in the detection, identification, and location of central and peripheral stimuli through three subtests increasing in visual attentional difficulty, as explained below.

1. Subtest 1 presents a car or truck silhouette of 2.0 × 1.5 cm inside a central rectangle in varying time intervals. The subject must identify, looking at the central rectangle (fixation box), if the figure shown is a car or a truck; therefore, this subtest assesses (in milliseconds) the visual processing speed.
2. Subtest 2 consists of the same task as Subtest 1 but adds the simultaneous localization of a peripheral object (car silhouette), which can be in eight different positions in cardinal and oblique axes. The peripheral object is shown in a random position at 11 cm (10.5º) from the central box in eight possible positions (0º, 45º, 90º, 135º, 180º, 225º, 270º, 315º). By looking at the central rectangle (and not moving the head or eyes), the subject must identify if the figure shown was a car or a truck and in which position the peripheral object appeared. Therefore, this subtest measures divided attention.
3. Subtest 3 consists of the same two previous tasks and adds visual distractors (47 triangles of the same size, color, and lighting of the car/truck silhouette), filling all the peripheral space. Therefore, the subject must identify if the central object is a car or a truck and choose in which position the peripheral object is presented, by filtering visual information. The addition of triangles considerably increases the task difficulty; therefore, this test measures selective attention.

**Fig. 2:**
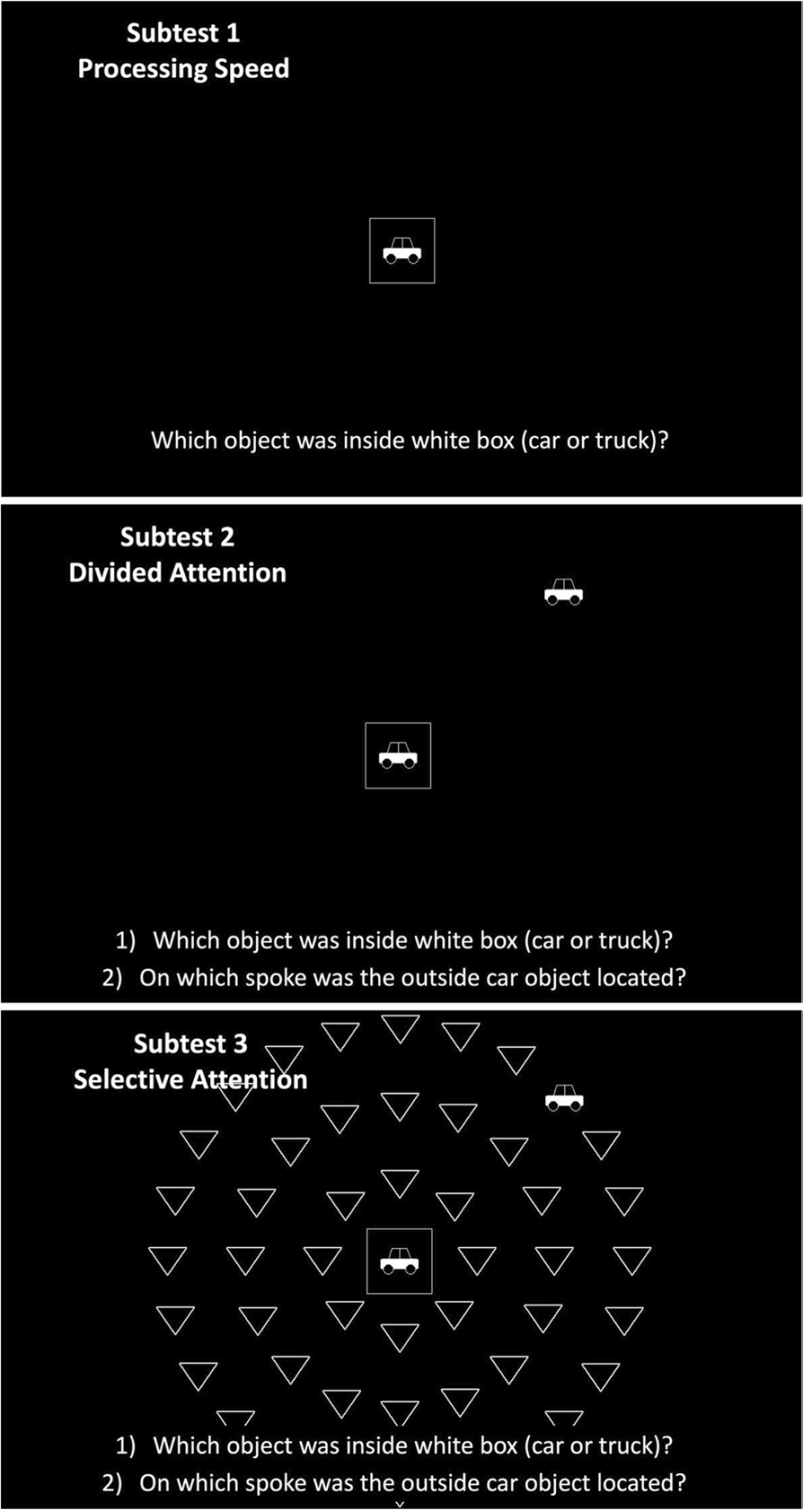
Subtest 1 (top), 2 (middle), and 3 (bottom) of the Useful Field of View (UFOV) test

The results of the test are defined as the duration in milliseconds in which the participant can correctly identify the stimulus. The duration of the image presentation varies between 17 and 500 milliseconds, using a double-staircase method to determine a threshold of 75% and obtain the visualization time. This test was performed by a weighted subsample of 75 healthy subjects (mean age of 50.3 ± 15.8 years) who accepted performing this additional test. Figure 2 presents pictures of each of the three subtests.

### Study participants

An a priori power analysis determined a minimum sample size of 110 subjects to obtain an effect size (*d*) of .695 (as obtained in a previous study [24]), a power of .95, and an α of .05. Using random, convenience, non-probability sampling [44], we approached and recruited subjects who fit the inclusion criteria from a psychological traffic assessment center and an optometric clinic in Valencia (Spain).

The inclusion criteria entirely comprised of subjects who 1) had not been involved in similar tests, 2) corrected distance visual acuity of 0.2 logMAR or better, 3) in case of using optical correction, this should be between − 6 and + 6 diopters both included, 4) had no oculomotor alterations, clinically significant crystalline opacification, nor health issues that may interfere with their reading or driving capacity, 5) were not taking medication nor were involved in medical treatments that could interfere with reading or driving ability, 6) had no ocular, cognitive, or neurological pathologies such as Alzheimer and Sclerosis, and 7) were not suffering from psychiatric disorders, nor had a history of substance abuse.

Therefore, 120 healthy Spaniards aged 20 to 85 years voluntarily participated in this study. Seventy-two (60%) of the participants were females and 48 males (40%). Sixty participants (50%) were drivers and 60 non-drivers. Included non-drivers were road users who had never driven a motor vehicle. All drivers had a driving license and at least 1.5 years of driving experience (for this reason, the minimum age is set to 20 years). The self-reported quality of life, quality of vision, and reading quality of the general sample were 2.85 ± .63, 2.79 ± .72, and 2.86 ± .75, respectively. This was measured on a scale with a maximum of 4 points (see “Main Questionnaire” section). Participants also answered the Visual Function test (VF14; see “Main Questionnaire” section); whose mean results were 96.56 ± 6.81 out of a maximum of 100 points (best results), with values ranging from 47.22 to 100.

Further features of the sample are presented in Table 1. The sample has been divided into three age groups according to young adults (≤ 35.00 years; 29.2%), adults (35.01–65.00 years; 48.3%), and older adults (≥ 65.01 years; 22.5%) to ease the interpretation of data. This age subdivision is based on previous research [45, 46].

**Table 1:** General characteristics of the sample divided into young adults (≤ 35 years), adults (35.01–65.00 years), and older adults (> 65 years)

Age group	Age Mean (SD)	Gender		Educational level			Status	
		Male	Female	Basic	Medium	Higher	Driver	Non-driver
Young adults *n* = 35	27.68 (4.04)	34.3%	65.7%	8.6%	11.4%	80.0%	51.4%	48.6%
Adults *n* = 58	55.57 (7.57)	43.1%	56.9%	46.6%	29.3%	24.1%	56.9%	43.1%
Older adults *n* = 27	70.98 (5.76)	40.7%	59.3%	57.7%	15.4%	26.9%	33.3%	66.7%
Total *n* = 120	50.90 (17.32)	40.0%	60.0%	37.8%	21.0%	41.2%	50.0%	50.0%

Age values are presented in years

**Fig. 3:**
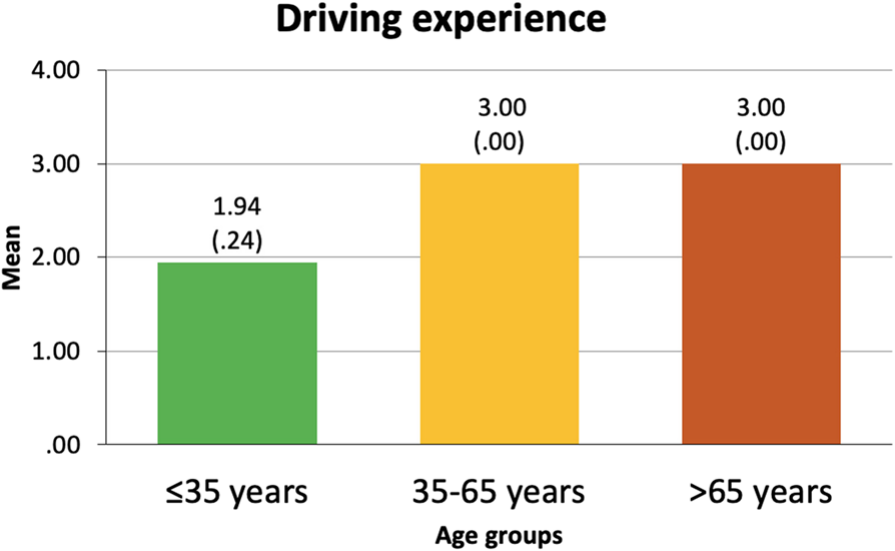
Driving experience of each of the three groups. Results are presented as mean and standard deviation. The Y-axis represents a categorical scale of 1 = less than 5 years; 2 = between 5 and 15 years; and 3 = more than 15 years

**Fig. 4:**
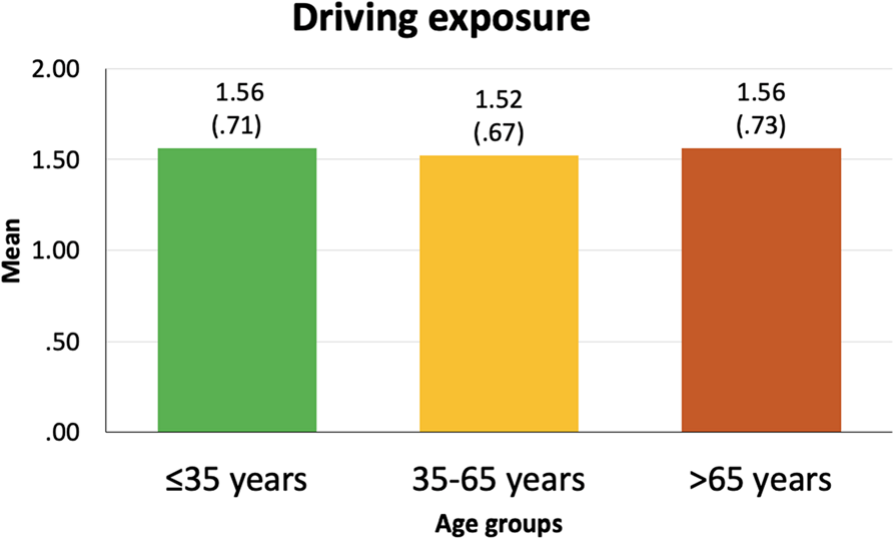
Driving exposure of each of the three age groups. Results are presented as mean and standard deviation. The Y-axis represents a categorical scale of 1 = under 2500 km/year, 2 = between 2500 and 10,000 km/year, and 3 = more than 10,000 km/year

**Fig. 5:**
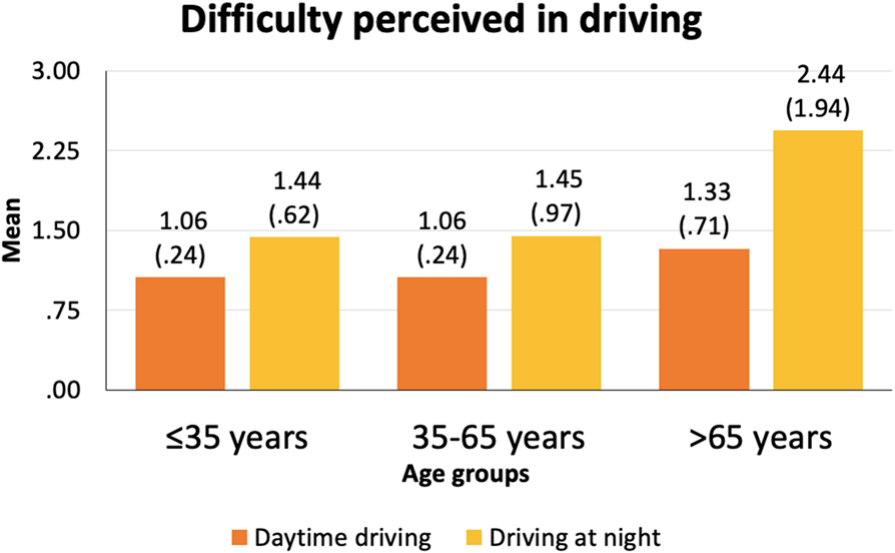
Difficulty perceived in daytime driving and driving at night of each of the three age groups. Results are presented as mean and standard deviation. The Y-axis represents a categorical scale between 0 (none) and 4 (much)

**Fig. 6:**
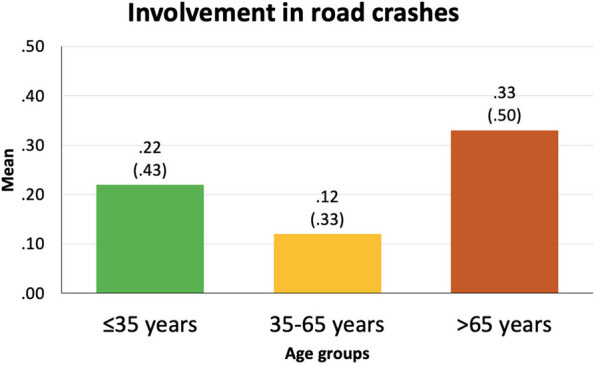
Involvement in road crashes in the last three years of each of the three age groups. Results are presented as mean and standard deviation. Possible results (Y-axis) are 0 (not involved) and 1 (involved). More specifically, the percentage of drivers who had been involved in a road crash in the last three years represented 21.1% of drivers under 35 years, 11.8% of drivers between 35 and 65 years, and 33.3% of drivers older than 65 years

Apart from the sample characteristics presented above, further drivers’ characteristics (i.e., driving experience, driving exposure, difficulty perceived in daytime driving and driving at night, and involvement in road crashes in the last three years) are specified hereunder (Figs. 3, 4, 5 and 6).

### Statistical analyses

The normality of the data distribution was tested using the Kolmogorov-Smirnov test. The results of both the ADEMd and UFOV showed a non-Gaussian distribution. Also, as frequently observed in test-based studies, the homoscedasticity assumptions could not be validated through Levene’s test. Therefore, and in light of the absence of multivariate normality, quantitative study variables were logarithmically transformed (following a log_10_ procedure); this procedure is useful to avoid statistical errors based on the variables’ skewness.

Furthermore, mean comparisons, useful to discriminate driver vs. non-driver scores in all tests, were conducted through robust Brown-Forsythe (B–F) tests, whose nature allows the application of statistical corrections to non-normal and heterogeneously distributed variables [47, 48]. Additionally, post hoc tests with Games-Howell adjustment, which do not assume homogeneity of variance, were performed for the age groups.

Moreover, bivariate correlation analysis (Spearman’s *rho*) analyzed the associations between pairs of variables (age and road safety skills). This correlational test was selected over Pearson’s (*r*) coefficients due to being more robust when the study involves sets of variables measured using an ordinal scale.

All the statistical analyses were performed with the statistical software IBM SPSS Statistics for Macintosh, Version 26.0 (Armonk, NY: IBM Corp). The a priori power analysis was performed with G*Power version 3.1.9.6 [49]. The cut-off criteria or significance level for this study was uniformly established at *p* < .050.

## Results

### General results of the ADEMd and UFOV

Before starting to analyze the results of the tests, it is important to bear in mind that both in the ADEMd (V_adj_, H_adj_, and Hd_adj_) and UFOV (Subtest 1, 2, and 3) the shorter the time, the better the test performance. On the other hand, the ADEMd ratios (H_adj_ / V_adj_; Hd_adj_ / V_adj_; Hd_adj_ / H_adj_) are constructs calculated ex-post (see “Procedures - ADEMd and UFOV” sections for further information). Table 2 presents the global descriptive results of the sample in both the ADEMd and UFOV.

In summary, significantly more time (*p* < .001) was required to complete each of the horizontal sheets of the ADEMd compared to the vertical one. The addition of distractors to the horizontal sheet significantly increased the time needed to complete the sheet (*p* < .001). Similarly, Subtest 1 of the UFOV (central vision) was completed in significantly less time (*p* < .001) than Subtest 2 (central and peripheral vision) and Subtest 3 (central and peripheral vision with distractors). Subtest 3 was the test of the UFOV with significantly (*p* < .001) the worst results.

**Table 2:** General results of the ADEMd and UFOV tests

	Variable	Range	Mean (SD)	P25	Median (P50)	P75
**ADEMd** (*n* = 120)						
** 1**	**V**_**adj**_	34.0–138.0	62.6 (18.1)	37.5	58.0	78.5
** 2**	**H**_**adj**_	30.0–165.0	66.7 (19.8)	42.2	62.0	81.8
** 3**	**Hd**_**adj**_	42.0–232.0	72.5 (23.9)	42.9	66.1	89.3
** 4**	**Ratio H**_**adj**_ **/ V**_**adj**_	.74–1.82	1.07 (.13)	.93	1.06	1.19
** 5**	**Ratio Hd**_**adj**_ **/ V**_**adj**_	.87 − 1.80	1.17 (.17)	.95	1.13	1.31
** 6**	**Ratio Hd**_**adj**_ **/ H**_**adj**_	.69–1.73	1.10 (.15)	.93	1.07	1.21
**UFOV** (*n* = 75)						
** 7**	**Subtest 1**	17.0–180.0	24.61 (23.40)	17.0	17.0	23.0
** 8**	**Subtest 2**	17.0–350.0	72.39 (83.56)	17.0	30.0	107.0
** 9**	**Subtest 3**	17.0–500.0	129.18 (123.26)	17.0	93.0	211.5

All values are expressed in seconds (Adult Developmental Eye Movement test [ADEMd]) or milliseconds (Useful Field of View test [UFOV])

*V* vertical sheets, *H* horizontal sheet, *Hd* horizontal sheet with distractors, *adj* adjusted time (considering the mistakes), *SD* standard deviation, *P* percentiles

### Correlation analysis

The main correlations evaluated are presented in Table 3. The most noteworthy findings are presented hereunder. Firstly, increased age in non-drivers was significantly associated with a worse visual function (VF14; *rho* = − .430) and worse performance in all the subtests of the ADEMd (V_adj_, H_adj_, and Hd_adj_; *rho* from .637 to .716) and UFOV (Subtests 1, 2, and 3; *rho* from .569 to .826).

Drivers only reported significant correlations between increased age and worse performance in the horizontal sheet with distractors (*rho* = .298) and all the subtests of the UFOV (*rho* from .548 to .838). Similarly, on the one hand, non-drivers presented significant negative correlations between reading hours and performance (more hours spent reading, better test performance) in all the ADEM subtests (*rho* from − .291 to − .363) and the Subtest 2 of the UFOV (*rho* = − .759).

**Table 3:** Correlation between the results of the ADEMd and UFOV tests

Variable			2	3	4	5	6	7	8	9
**1**	**Age**	Non-driver	− .368^**^	− .430^**^	.639^**^	.637^**^	.716^**^	.569^*^	.637^*^	.826^**^
		Driver	− .291^*^	− .117	.069	.126	.298^*^	.548^**^	.717^**^	.838^**^
**2**	**Hours reading**	Non-driver	-	.118	− .361^**^	− .291^*^	− .363^**^	− .422	− .759^*^	− .617
		Driver	-	.062	− .057	− .079	− .168	− .178	− .435^**^	− .386^**^
**3**	**VF-14**	Non-driver		-	− .520^**^	− .515^**^	− .392^**^	− .363	− .397	− .329
		Driver		-	− .327^*^	− .268^*^	− .285^*^	− .011	− .167	− .230
**4**	**ADEMd V**_**adj**_	Non-driver			-	.932^**^	.872^**^	− .018	− .053	− .090
		Driver			-	.787^**^	.707^**^	.137	.243	.206
**5**	**ADEMd H**_**adj**_	Non-driver				-	.888^**^	.032	.175	.140
		Driver				-	.787^**^	.059	.163	.307^*^
**6**	**ADEMd Hd**_**adj**_	Non-driver					-	.349	.256	.334
		Driver					-	.322^*^	.350^**^	.410^**^
**7**	**UFOV Subtest 1**	Non-driver						-	.710^**^	.495
		Driver						-	.550^**^	.457^**^
**8**	**UFOV Subtest 2**	Non-driver							-	.703^**^
		Driver							-	.816^**^
**9**	**UFOV Subtest 3**	Non-driver								-
		Driver								-

*VF-14* Visual Function Index Test, *ADEMd* Adult Developmental Eye Movement test with distractors, *UFOV* Useful Field of View test, *V* vertical sheets, *H* horizontal sheet, *Hd* horizontal sheet with distractors, *adj* adjusted time (considering participants’ mistakes during the testing phase)

^**^Correlation is significant at the *p* < .001 level (2-tailed)

^*^Correlation is significant at the *p* < .05 level (2-tailed)

On the other hand, drivers only presented significant negative correlations between reading hours and performance in Subtests 2 and 3 of the UFOV (*rho* from − .386 to − .435). Higher scores in the VF-14 (better visual function) were negatively and significantly correlated (better test performance) with all the ADEMd parameters in both non-drivers (*rho*_*s*_ ranging from − .392 to − .520) and drivers (*rho ranging* from − .268 to − .327).

Finally, it is worth highlighting that correlations between H_adj_ and Subtest 3 of the UFOV (worse results in H_adj_ entail worse results in Subtest 3; *rho* .307) and between Hd_adj_ and all the three subtests of the UFOV (worse results in Hd_adj_ entail worse results in the UFOV; *rho* from .322 to .410) were found significant only among the group of drivers.

### Gender-based comparisons

Regarding between-gender comparisons (conducted using male/female participant labels as discrete categories), it was found that, overall, significantly worse results were observed for females compared to males in the ADEMd. Moreover, no significant differences were observed between male and female participants in terms of UFOV performance. Descriptive data and the full set of inferential analyses are described below, in Table 4.

**Table 4:** Descriptive data and comparisons between genders

Variable	Gender	Mean	SD	95% CI		F test (Brown-Forsythe)	
				Lower	Upper	Stat.	Sig.
**ADEMd**							
** V**_**adj**_	1. Female	66.21	19.77	61.56	70.86	8.74	**
	2. Male	57.18	13.68	53.21	61.15		
** H**_**adj**_	1. Female	70.07	21.85	64.93	75.20	6.25	*
	2. Male	61.66	15.01	57.30	66.02		
** Hd**_**adj**_	1. Female	76.34	27.56	69.87	82.81	5.76	*
	2. Male	66.83	15.72	62.26	71.40		
**UFOV**							
** Subtest 1**	1. Female	24.05	26.88	15.22	32.89	.03	^N/S^
	2. Male	24.97	19.56	18.45	31.49		
** Subtest 2**	1. Female	64.70	83.62	36.82	92.58	.623	^N/S^
	2. Male	80.08	83.92	52.10	108.06		
** Subtest 3**	1. Female	107.75	114.24	69.10	146.40	2.19	^N/S^
	2. Male	150.03	129.58	106.82	193.23		

All values are expressed in seconds (Adult Developmental Eye Movement test [ADEMd]) or milliseconds (Useful Field of View test [UFOV])

*V* vertical sheets, *H* horizontal sheet, *Hd* horizontal sheet with distractors, *adj* adjusted time (considering the mistakes), *SD* standard deviation, *Stat.* Brow-Forsythe statistic, *Sig.* *p*-value of significance

^N/S^Non-significant difference

^*^significant at *p* < .05

^**^significant at *p* < .01

### Comparisons among age groups

As for age comparisons, it is worth first highlighting that all the subjects in the youngest group obtained the best possible result in Subtest 1 (17.00 milliseconds). However, no significant differences were observed between the adults and older adults. The best results in all the rest of the parameters were obtained by young adults, followed by adults, and the poorer performance results were obtained by older adults (see Table 5).

**Table 5:** Descriptive data and comparisons between age groups

Variable	Age group	Mean	SD	95% CI		F test (Brown-Forsythe)		Post-hoc	
				Lower	Upper	Stat.	Sig.	Gr.	Sig.
**ADEMd**									
** V**_**adj**_	1. Young adults	53.35	7.85	50.66	56.05	14.65	***	1–2	**
	2. Adults	61.85	17.34	57.29	66.41			1–3	***
	3. Older adults	76.18	21.17	67.80	84.55			2–3	**
** H**_**adj**_	1. Young adults	57.13	7.36	54.61	59.66	10.87	***	1–2	**
	2. Adults	66.21	18.00	61.48	70.95			1–3	***
	3. Older adults	80.16	26.40	69.72	90.60			2–3	*
** Hd**_**adj**_	1. Young adults	58.88	9.30	55.69	62.07	13.23	***	1–2	***
	2. Adults	72.57	19.14	67.54	77.60			1–3	***
	3. Older adults	90.16	33.63	76.86	103.47			2–3	*
**UFOV**									
** Subtest 1**	1. Young adults	17.00	.00	17.00	17.00	3.44	*	1–2	^N/S^
	2. Adults	24.49	25.77	16.35	32.62			1–3	^N/S^
	3. Older adults	38.33	30.80	18.76	57.90			2–3	^N/S^
** Subtest 2**	1. Young adults	18.52	4.81	16.33	20.71	15.53	***	1–2	***
	2. Adults	74.07	75.50	50.24	97.91			1–3	**
	3. Older adults	160.92	107.05	92.90	228.93			2–3	*
** Subtest 3**	1. Young adults	22.90	15.68	15.56	30.24	32.22	***	1–2	***
	2. Adults	134.98	102.51	102.62	167.33			1–3	***
	3. Older adults	286.50	114.63	213.67	359.33			2–3	**

All values are expressed in seconds (Adult Developmental Eye Movement test [ADEMd]) or milliseconds (Useful Field of View test [UFOV])

*V* vertical sheets, *H* horizontal sheet, *Hd* horizontal sheet with distractors, *adj* adjusted time (considering the mistakes), *SD* standard deviation, *Stat.* Brow-Forsythe statistic, *Sig.* *p*-value of significance, *Gr.* Groups being compared (1–2: Young adults with adults; 1–3: young adults with older adults; 2–3: adults with older adults)

^N/S^Non-significant difference

^*^significant at the level *p* < .050

^**^significant at the level *p* < .010

^***^significant at the level *p* < .001

### Comparison between drivers and non-drivers

Regarding comparisons between drivers and non-drivers, while highly significant differences were observed in all the ADEMd parameters, no significant differences existed in the performance of the UFOV. The results can be found in Table 6.

**Table 6:** Descriptive data and comparisons between drivers and non-drivers

Variable	Status	Mean	SD	95% CI		F test (Brown-Forsythe)	
				Lower	Upper	Stat.	Sig.
**ADEMd**							
** V**_**adj**_	1. Non-driver	70.12	20.38	64.86	75.39	24.98	***
	2. Driver	55.07	11.34	52.14	58.00		
** H**_**adj**_	1. Non-driver	73.50	23.10	67.54	79.47	15.98	***
	2. Driver	59.90	12.68	56.63	63.18		
** Hd**_**adj**_	1. Non-driver	79.51	29.08	71.99	87.02	11.04	***
	2. Driver	65.56	14.50	61.82	69.31		
**UFOV**							
** Subtest 1**	1. Non-driver	30.00	41.77	6.87	53.13	.39	^N/S^
	2. Driver	23.13	16.23	18.94	27.32		
** Subtest 2**	1. Non-driver	72.80	95.29	20.03	125.57	.00	^N/S^
	2. Driver	72.29	81.21	51.12	93.45		
** Subtest 3**	1. Non-driver	130.53	113.27	67.81	193.26	.00	^N/S^
	2. Driver	128.83	126.64	95.53	162.13		

All values are expressed in seconds (Adult Developmental Eye Movement test [ADEMd]) or milliseconds (Useful Field of View test [UFOV])

*V* vertical sheets, *H* horizontal sheet, *Hd* horizontal sheet with distractors, *adj* adjusted time (considering the mistakes), *SD* standard deviation, *CI* confidence interval, *Stat.* Brown-Forsythe statistic

^N/S^Non-significant difference

^***^significant at the level *p* < .001

## Discussion

This study aimed to assess the potential of the Adult Developmental Eye Movement with a distractors test (ADEMd) to differentiate between drivers and non-drivers, and to compare the results with the UFOV test. A previous version of the ADEMd, the Adult Developmental Eye Movement test (ADEM), has proven valid for measuring oculomotor efficiency and visual attention in drivers and non-drivers [24]. In this regard, better results were obtained for drivers compared to non-drivers in the ADEMd, but non-significant differences were encountered in the UFOV, which partially confirms the study hypothesis.

The new results presented in this study suggest that the addition of distractors in the ADEMd enhances the assessment potential of the test concerning visual processing speed and divided and selective attention, with these abilities being relevant for proper driving [27, 50, 51]. This can be seen in the positive correlation encountered only in the drivers between the horizontal time with distractors (Hd_adj_) and the three subtests of the UFOV (see Table 3). Oppositely, the vertical time (without distractors, V_adj_) was not correlated with the UFOV subtests, and the horizontal time without distractors (H_adj_) was only correlated with Subtest 3 (selective attention), again only in drivers. It should be noted that the significant correlations encountered between the ADEMd and UFOV ranged between (*rho*) .307 and .410, which are considered low to moderate correlations [52, 53].

Differences between the UFOV and ADEMd could lie in the possibility of performing ocular movements or not within the test protocols. In this regard, while ocular movements are allowed to perform the ADEMd, the head or the eyes should not move to perform the UFOV. Therefore, we could state that, although the addition of distractors approaches the ADEMd to the UFOV compared to the ADEM (without distractors), the tests are measuring different constructs (see “ADEMd” and “UFOV” sections) and, therefore, should not be interchangeably used.

Taken together, these results suggest that the ADEMd could be useful for identifying deficits in visual processes, cognitive automaticity, and divided attention in drivers, being these parameters commonly assessed during eyesight examinations necessary to obtain or renew one’s driving license [9, 16, 54]. Although we did not directly measure this in the present study, some literature sources suggest that the relevance of ocular movements evaluation could lie in understanding drivers’ visual strategies and processes used for determining their visual information management and predicting their driving performance [13, 26, 55]. Future studies should assess the relationship between the visual strategies employed to complete the ADEMd and those employed in driving situations, either in a monitored vehicle or simulators.

Considering what has been mentioned above, the outcomes of this research, in light of other empirical experiences, are worth discussing to draw conclusions. Hereunder, the results of the present study are discussed. Firstly, the correlations and then, the between-group comparisons (gender, age, and driver condition) will be interpreted and discussed.

### Test correlations

The most remarkable finding regarding the correlations was that Hd_adj_ highly correlated significantly with all three subtests of the UFOV only in drivers. Although not including ocular movements, negative results in Subtests 1 (processing) and 2 (divided attention) of the UFOV are associated with poorer driving performance in specific maneuvers, such as turning and merging onto a road [18, 19, 56]. These two subtests of the UFOV significantly correlated with the horizontal sheet with distractors (Hd_adj_-Subtest 1: *rho*: .322, *p* < .05; Hd_adj_-Subtest 2: *rho*: .350, *p* < .001). Subtest 3 of the UFOV (selective attention) is associated with worse results in all driving measurements [20]. This subtest of the UFOV significantly correlated with both horizontal sheets of the ADEMd (Hd_adj_: *rho* .410, *p* < .001; H_adj_: *rho* .307, *p* < .05). With these results in mind, future studies using whether naturalistic or simulated driving situations, should assess the potential associations between the ADEMd scores and the actual driving performance of individuals.

As shown in Table 3, age negatively affects non-drivers’ performance in the ADEMd and UFOV, but drivers’ performance is only involved in the UFOV and the Hd_adj_ of the ADEMd. It is worth highlighting that non-significant correlations were encountered between the age of drivers and the vertical and horizontal sheets of the ADEMd (both sheets without distractors). This could be due to distracting elements’ influence on older drivers [57, 58]. Another relevant factor was that reading hours seemed to positively affect non-drivers’ performance in the ADEMd, but not that of drivers (non-significant association encountered). A potential explanation for these results is that driving entails certain oculomotor and cognitive training, which are relevant for reading performance [24, 33, 59].

Lastly, and regarding bivariate analysis outcomes, it is worth highlighting that the VF-14 significantly correlated with the ADEMd. This result is in line with previous literature that encountered the VF-14 to be a useful indicator of oculomotor function [60]. According to previous literature [20], the correlation between the VF-14 and the UFOV was non-significant. This suggests that these tests may measure different aspects of visual function and should be used together to obtain a comprehensive assessment of visual function.

### Gender differences

The first takeaway from this set of analyses was that, while significant differences appeared between genders in all three sheets of the ADEMd (worst results for females), non-significant differences were observed in the UFOV. This is in contrast with previous expert literature, which did not find significant between-gender differences in children’s DEM performance [61]. Although in the review of Logan & Johnston [62], several authors associate the between-gender differences in reading with variations in brain activation patterns, caution should be applied with such approaches [63, 64], and focus should be placed on the reading strategies adopted by subjects of both genders.

In this regard, one potential literature-based explanation could be the more cautious behavior of females compared to males applicable to different risk-related spheres [12, 65, 66]. This is theoretically feasible in consideration of studies suggesting that females usually employ a “more precise than fast” visual scanning strategy. Therefore, it makes sense to find them as those reporting fewer mistakes, but a greater average global time to perform the task [62]. In addition, previous large-scale studies have argued that visual issues are more frequent among females, coherently to the case of the general population [12], something that could influence the results, despite not being objectively controlled in this study, in which the visual health “filters” were basically self-reported.

### Comparisons among age groups

Older participants obtained significantly worse results in all three sheets of the ADEMd and the subtests of the UFOV focused on divided and selective attention. This could be expected considering the previous expert literature reporting that being aged below 30 seems to guarantee a better precision of performance, as well as accuracy in detecting visual targets through saccadic eye movements [10]. On the other hand, older subjects present a decline in ocular searching [10, 16, 54] and reading speed [67, 68], visual attention [69], and are more affected by distractors [70–72]. Also, in this sense, older adults have been reported to read more carefully compared to younger readers, making shorter forward eye movements and fixating closer to the beginnings of two-character target words in sentences [67]. This could also be a reason for trying to minimize the mistakes, obtaining a greater total time. On the other hand, the non-significant differences between age groups in the Subtest 1 of the UFOV could be that peripheral vision is relatively stable through aging [73]. However, there is a certain controversy in this regard.

Another potential explanation might be the older subjects not maintaining a fixed gaze, instead glancing at the targets to solve Subtest 1 of the UFOV and, therefore, obtaining similar results compared to younger subjects, as reported in a previous study [74]. Considering that divided and selective attention is required in the other two subtests of the UFOV, older subjects obtain significantly worse results than younger ones [75]. These age-related differences are in line with previous expert research on the ADEM (without distractors) that proposed age as a performance predictor [23–25].

Although our study does not directly assess driving behaviors, the implications of these results could be linked to driving in a certain manner. Given the observed worse test performance, older individuals typically require more fixation time and have slower saccades compared to younger individuals to navigate the same driving scene [10, 50]. This underscores the consideration of older drivers as a potentially higher-risk group [76]. Future studies associating the ADEMd with real-driving performance would be necessary to draw robust conclusions in this regard.

### Comparison between drivers and non-drivers

Concerning the comparison between drivers and non-drivers, it is worth highlighting that drivers obtained significantly better results in all the sheets of the ADEMd and that non-significant differences were encountered between drivers and non-drivers in any of the three subtests of the UFOV. This could be due to the differences in the test procedures. While in the ADEMd, participants are allowed to move their eyes, in the UFOV the gaze should be fixed on a central point. These better results for drivers could, therefore, be associated with the hypothesis of drivers gaining certain oculomotor training while performing the driving task, with drivers using overt (with fixations) and covert (without fixations) attentional strategies more efficiently than non-drivers to detect hazards [16].

Also, previous research that analyzed the carryover effect of eye movements between two tasks reported that non-drivers had worse results than drivers [33]. Similarly, inexperienced drivers have been associated with longer fixations [16] and more focus on central vision [9].

### Limitations of the study and further research

Although all the procedures were carefully designed and conducted, there are several limitations and future research lines to mention. Firstly, it is worth highlighting that the ADEMd indirectly evaluates the results of the oculomotor function and reading speed. However, this is one of several possible data sources and complementary information that could be useful to cross-check these results. In this regard, future studies would find it useful to corroborate the hypotheses tested in the present study with the use of an eye-tracker device and other visual functioning tests.

Secondly, although a posthoc power analysis with a correlation coefficient of 0.337 and an alpha of 0.05 resulted in a power of 0.91, and this gives good statistical background to the study, it should be noted that the UFOV test was still administered to a relatively small sample (*n* = 75) due to instrument and time constraints, and more cases could enrich both qualitatively and quantitatively potential research outcomes using it.

Finally, and given that this study does not further explore the external validity of the findings, but rather tests its assumptions from a cross-sectional (single-measure and method) perspective, the findings of the present study should be cautiously understood until the tests conducted are applied in a driving environment (whether of a simulated or naturalistic nature) through a further study. Additionally, attention should be paid to potential confounding factors not commonly in experimental studies, including those of subjective nature, e.g., motives for engaging in driving, or the reasons for the non-drivers being non-drivers.

With this approach in mind, it would be interesting to compare the ADEMd test performance between drivers who drive safely and those who perform unsafely, either in a monitored vehicle or a driving simulator. Therefore, the results presented in this study must be interpreted with caution until more scientific evidence evaluates the direct correlation between the performance in the ADEMd and driving outcomes or neurodegenerative conditions. With this, future research may assess the viability of restricting less skilled drivers to use routes with greater risk exposure (e.g., high-speed highways, and reduced visibility situations).

## Conclusion

In summary, the results of this study support the hypothesis that drivers obtain better performance in visual-verbal tests (ADEMd) compared to non-drivers, with the addition of distractors directly relating to a well-known, validated test for drivers (UFOV) that assesses visual processing.

Considering all the discussed outcomes, the ADEMd could be of interest to indirectly evaluate parameters, such as oculomotor function and visual attention. Therefore, and while more research is still required in this regard (this is just the first empirical experience, and more insights are needed), the ADEMd has shown potential to be a useful and inexpensive tool to increase and optimize the data gathered in terms of oculomotor and cognitive function of drivers.

## Data Availability

The datasets used and/or analyzed during the current study are available from the corresponding author upon reasonable request.

## Abbreviations

ADEM: Adult Developmental Eye Movement test
ADEMd: Adult Developmental Eye Movement with Distractors test
DEM: Developmental Eye Movement test
UFOV: Useful Field of View test
